# Investigating estimand considerations in adaptive trials: a systematic review

**DOI:** 10.1186/s13063-026-09490-0

**Published:** 2026-02-06

**Authors:** Fran Piazza, Hannah Wallace, Rachel Phillips, Suzie Cro, Zohra Zenasni

**Affiliations:** Imperial Clinical Trials Unit, School of Public Health, Imperial College London, London, UK

**Keywords:** Randomised controlled trials, Adaptive, Trials, Estimands, Systematic review, Protocols, Statistical analysis plans

## Abstract

**Background:**

Randomised controlled trials (RCTs) are the gold standard for evaluating treatment effects, with the results informing policy and clinical practice. To ensure appropriate methods are utilised and to avoid misinterpretation of the results of a clinical trial, it is vital that we understand the research question a trial aims to answer. However, there is often ambiguity in how trialists define their research questions. In 2019, an addendum to the international trial regulatory guidelines (ICH E9 (R1)) introduced the estimand framework to combat this. A review of protocols published in 2020 investigated the early adoption of the estimand framework and found no uptake as well as a lack of clarity on key items such as the handling of intercurrent events. The aim of this review was to identify the current application of the estimand framework specifically to trials with an adaptive design.

**Methods:**

The search strategy aimed to identify trial protocols and statistical analysis plans that described RCTs published in two journals (BMJ Open and Trials) in 2023. Articles were eligible if they related to phase 2–4 trials with an adaptive design. A pre-piloted data extract form was used to extract data relating to study details, intercurrent events and estimands.

**Results:**

One thousand five hundred and forty-one articles were identified by the initial search. Following screening, 146 articles were identified as meeting the eligibility criteria. Of the eligible articles, five (3%) stated their primary estimand, and of these, three (2%) stated all five estimand attributes. Ninety-four (64%) articles described one or more intercurrent events; these included a total of two hundred and thirty-two intercurrent events described. Fifty-two (36%) articles did not describe any intercurrent events. No articles specified the estimand for any planned interim analyses or considered the implications of adaptations on the primary estimand.

**Conclusions:**

This review provides evidence that there is still a lack of uptake of the estimand framework in RCTs. Wider application of the estimand framework would ensure clarity in the reporting and interpretation of clinical trial results. In addition, clear guidance on how to implement the estimand framework to trials with an adaptive design is needed.

**Supplementary Information:**

The online version contains supplementary material available at 10.1186/s13063-026-09490-0.

## Background

In the healthcare setting, randomised controlled trials (RCTs) are the gold standard for evaluating treatment effects with the results informing policy and clinical practice. To ensure appropriate methods are utilised (the trial’s design, conduct and analysis) and to avoid misinterpretation of the results of a trial, it is vital that we understand the research question a trial aims to answer [1]. However, there is often ambiguity in understanding the precise research question a trial aims to address.

The estimand framework was formally introduced in a 2019 addendum to the international trial regulatory guidelines (ICH E9 (R1)) with the aim of ensuring that the research question a trial intends to investigate is precisely described at the outset [2]. The trial design, conduct and analysis can then be planned to align with the estimand(s) of interest to ensure the trial addresses what is of most relevance.

There are five key attributes that need to be specified to define the estimand of interest (Table 1 describes these). One of these attributes includes specifying important post-randomisation events that can affect either the interpretation or existence of the outcome of interest, referred to as intercurrent events, and the strategy to handle them. Different strategies for handling intercurrent events are required to answer different questions and can result in differing conclusions about the treatment effect. For example, a treatment policy strategy assumes the intercurrent event is considered part of the treatment condition and addresses the treatment effect, regardless of the intercurrent event. Alternatively, a composite strategy incorporates the intercurrent event into the endpoint definition. The different strategies that can be used are detailed further in [3].

**Table 1:** Five key attributes of the estimand framework with an applied example [3, 4]

Attribute	Definition	Example taken from Stark et al. [5]
Population	The wider group of patients the treatment effect is being estimated for	“infants born less than 28 weeks’ gestation who require a RBC transfusion”
Treatment conditions	The different intervention strategies being compared	“the investigational RBC transfusion intervention product, regardless of adherence to the study protocol (including receipt of the wrong allocated study product) and exposure to other transfusion products and regardless of intervention discontinuation (treatment policy strategy)”^*^
Outcome (or endpoint)	The measurement taken from each participant to assess the treatment effect on	“incidence of any one or more of the following prior to first discharge home: all-cause mortality, BPD (assessed on a physiological basis at 36 weeks post- menstrual age), ROP grade > 2 (uni- or bilateral), NEC Bell’s stage ≥ 2”
Summary measure	The statistic calculated to compare the outcome between treatment conditions	“relative risk of composite of major neonatal morbidity and/or mortality between treatment groups (transfusion with washed RBCs vs transfusion with unwashed RBCs)”
Intercurrent events and strategy to handle each	Each intercurrent event, which are the events that occur in a trial after randomisation and affect either the interpretation or existence of an individual’s outcome and the strategy used to handle each	Death: “addressed by the endpoint definition (composite variable strategy).” Non-adherence to study protocols (including receiving the wrong study product), exposure to other transfusion products and intervention discontinuation: “are all addressed by the treatment condition of interest attribute (treatment policy strategy).”

Acronyms: *RBC* red blood count, *BPD* bronchopulmonary dysplasia, *ROP* retinopathy of prematurity, *NEC* necrotising enterocolitis

^*^The original article did not specify the control condition as part of the estimand

A review of protocols for RCTs published in Trials and BMJ Open in 2020 examined early uptake of the estimand framework and found no RCTs explicitly defined the estimand [6]. A further review examined uptake of the estimand framework in RCT results articles published in 2020 in six high-impact medical journals and similarly identified that the precise treatment effect being investigated in most trials is unclear, mainly due to a lack of clarity on the approach to handling intercurrent events [7]. The authors determined whether each attribute of the estimand (Table 1) was at the very least inferable even if it wasn’t explicitly stated. Out of 255 eligible articles included in the review, none stated all five attributes of the estimand. Of the 242 (95%) trials that reported an intercurrent event, how it was handled in the analysis could be inferred in only 125 cases (49%) [7].

The importance of the estimand framework is demonstrated in an example reported by Kahan et al. [6]. The original paper describes a trial of cabazitaxel in patients with metastatic prostate cancer which found a significant improvement in the quality of life of participants as assessed by the EQ-5D [8]. Whilst readers of the original article might assume that the reported results represent the benefit of cabazitaxel if introduced as part of usual care, on closer inspection Kahan et al. demonstrated that the result presented represented the treatment effect in the hypothetical setting where men with metastatic prostate cancer never experience disease progression or death [6]. The estimand framework aims to eliminate any ambiguity in the research question being addressed by ensuring greater specificity.

Another area of growth in clinical trials is the implementation of trials with adaptive designs. Adaptive designs allow researchers to make pre-planned changes to how the trial is conducted once the study is underway, without undermining its validity and integrity [9]. Such trials can allow for uncertainties at the point of design and are becoming more popular as they can provide a more efficient means to establish if treatments are safe and effective [10]. Adaptations are often triggered by pre-planned interim analysis which analyses the data during the trial. There are many elements of a trial that can be adapted.

ICH E9 (R1) [2] and the FDA guidance for adaptive design trials [11] do not mention estimands for adaptive designs. The CONSORT guidelines for adaptive designs advise trialists that they should report adaptive trial methods and results, in line with the prespecified estimand for both interim and final analyses [12]. Trial adaptations can have important implications on the estimand attributes [13]. Adaptations may cause the primary estimand to change, e.g. population enrichment or multi-arm, multi-stage (MAMS) and platform designs where interventions can be dropped and added as the trial progresses. Group sequential designs (GSDs) and sample size re-estimation shouldn’t impact the primary estimand, but it is important to specify the interim estimand. Firstly, because an interim analysis could become the final analysis in a GSD, and secondly, it is important to clarify whether the primary estimand is identical for interims and final analyses or whether attributes and the question being asked change between the interim and final analyses.

The aim of this research was to identify current application of the estimand framework specifically to trials with an adaptive design, focusing on trials that included adaptive randomisation, sample size re-estimation, adding and dropping interventions (e.g. MAMS trials or platform trials) and GSDs with planned stopping rules.

## Methods

A full protocol was specified prior to undertaking the search and is included in Additional file 1; Appendix 1.

### Search strategy

The search strategy aimed to identify published trial protocols and statistical analysis plans (SAPs) that described RCTs in humans published in the year 2023 in two medical journals known to publish trial protocols and SAPs: BMJ Open and Trials. To identify articles describing RCTs, we searched for the terms: ‘randomised’, ‘randomized’, or ‘randomly’ or ‘placebo’ in article titles and abstracts or articles with a publication type of controlled clinical trial. We excluded articles with ‘systematic review’ or ‘meta-analysis’ in the title. The search was performed using the Medline and Embase databases via OvidSP. The full search strategy is included in Additional file 1: Appendix 2. The final search was conducted in July 2024.

### Eligibility criteria

Full text protocols, or SAPs, published in either the BMJ Open or Trials in 2023 describing phase 2–4 adaptive RCTs in humans were eligible for inclusion. Trials with any of the following adaptive elements were eligible for inclusion: planned addition or dropping of arms, formal stopping rules, sample size re-estimation or adaptive randomisation. Pilot, feasibility, phase I and non-randomised controlled trials were excluded as the use of estimands may be different. Letters, commentaries, systematic reviews and trials with cost-effectiveness as their primary outcome were also excluded.

### Selection process

All search results were first imported into Endnote where duplicates were removed, all remaining articles were screened for eligibility using the Covidence web-based tool. Title and abstract screening were done by two reviewers (FP, HW); initially, articles were screened for eligibility by both reviewers to ensure consensus; disagreements were resolved by discussion with a third reviewer where necessary (ZZ or RP). Once agreement was reached, the remaining articles were divided across reviewers and screened individually. Articles passing initial screening underwent full-text review; again, initially, this was undertaken by two reviewers (FP, HW) to ensure consensus, with disagreements resolved in discussion with a third reviewer. Once consensus was reached, the remaining articles were divided across reviewers and screened individually. During full-text screening, it was decided to further exclude articles that did not include the words ‘adaptive’, ‘re-estimation’ or ‘interim’ throughout the article.

### Data extraction

Data was extracted into a pre-piloted standardised data extraction form (see Additional file 2) by one reviewer (FP or HW). Data was extracted on study details as well as whether the primary and interim estimands were specified. We collected information on intercurrent events, specifically whether they were explicitly or not explicitly stated. If intercurrent events were stated, we collected data on how they would be handled in the analysis. Data from protocols and SAPs that specified an estimand were extracted by both reviewers to ensure agreement. We referred to the supplementary material for additional details only when referred to from the main article.

### Outcomes

For the primary outcome, we recorded whether each of the five estimand attributes (population, treatment condition, summary measure, outcome variable and intercurrent events—Table 1) was explicitly or not explicitly described, the details for each and where these details were reported (main text or supplementary material when referred to). Intercurrent events were recorded along with any prespecified plans for handling them in the analysis and the strategy this aligned with if explicitly described as part of the estimand. For the outcome variable, we also recorded if it was a composite variable that incorporated an intercurrent event. We also extracted the planned methods of statistical analysis.

Trials have typically always reported events such as treatment discontinuation, use of rescue therapy and death but only with the advent of the ICH E9 (R1) addendum have trialists begun to consistently refer to such events as intercurrent events. Therefore, to gain an understanding of how many trials’ handling of intercurrent events would be relevant for, we also recorded any post-randomisation events that were reported regardless of whether this was in the context of the estimand framework.

### Statistical methods for data synthesis

We used frequencies and percentages to summarise the data. Two subgroup analyses were performed comparing descriptive statistics according to the following: sponsorship—academic/not-for-profit or pharmaceutical/for-profit sponsors; and article type—SAP or protocol. Analysis was performed using RStudio version 2024.09.0 + 375.

## Results

### Search results and trial characteristics

**Fig. 1:**
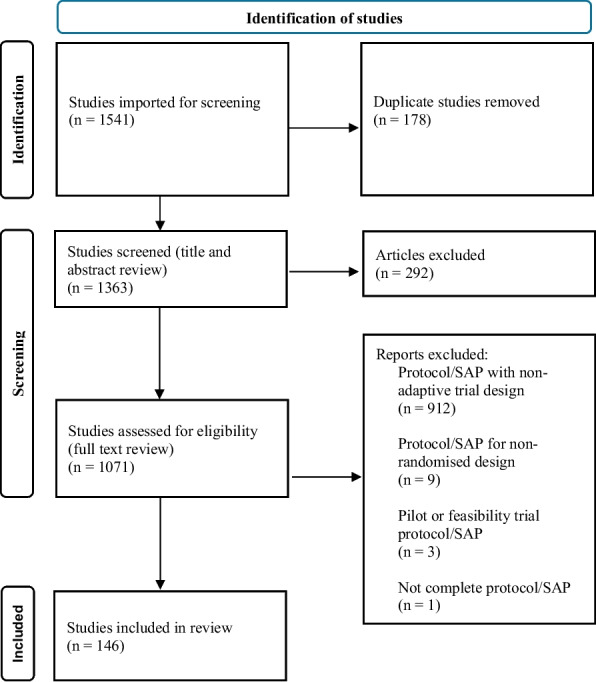
PRISMA diagram

Out of the 1541 articles identified by our initial search, 146 fulfilled the predefined inclusion criteria (Fig. 1). Eighty-six (59%) of the eligible articles were published in Trials and 60 (41%) published in BMJ Open. Protocols were the predominant article type (139, 95%) compared to only 6 (4%) SAPs, and one (1%) combined protocol and SAP publication (Additional file 3: Appendix 3 Table 1). Most trials had an academic or not-for-profit sponsor (120, 82%); 10 (7%) had a pharmaceutical or for-profit sponsor and 16 (11%) sponsors were not specified (Additional file 3: Appendix 3 Table 1).

The most common adaptive element was a planned stopping rule (116, 78%); 33 (22%) had planned sample size re-estimation; 12 (8%) used adaptive randomization; and 8 (5%) had planned addition of or dropping of arms (Additional file 3: Appendix 3 Table 2). Further details on trial characteristics can be found in Additional file 3: Appendix 3 Table 2.

### Intercurrent events

A total of 94 (64%) articles described intercurrent events either explicitly using the intercurrent event term or not explicitly (Table 2). Of these, eighty-nine (60%) articles discussed events that would be considered intercurrent events under ICH E9 (R1), without explicitly labelling them as such, i.e. ‘discontinuation criteria’. Across the 94 (64%) articles where intercurrent events were described (either explicitly or not explicitly), there were 232 events. There was a fairly even spread of intercurrent event types with the most common being treatment discontinuation (124, 53%). Over a third (52, 36%) of articles did not describe any intercurrent events.

**Table 2:** Intercurrent events

Characteristics	No. of trials (*n* = 146)	%
Intercurrent event described		
No	52	36
Yes explicitly	5	3
Yes, not explicitly	89	61
Type of intercurrent event described^*^		
Treatment non-adherence with no reason	22	9
Treatment non-adherence due to AE	26	11
Treatment non-adherence with reason (not AE)	31	13
Treatment discontinuation with no reason	40	17
Treatment discontinuation due to AE	46	20
Treatment discontinuation with reason (not AE)	38	16
Use of additional treatment not part of usual care (e.g. rescue therapy)	8	3
Treatment switching	3	1
Death	15	6
Other	3	1

^*^Articles could describe multiple events

### Primary estimand

One hundred and forty-one (97%) articles did not state their primary estimand. Five articles (3%) stated their primary estimand; only three (2%) fully stated their primary estimand including all five estimand attributes, whilst two articles (1%) partially stated their primary estimand (Table 3) [5, 14–17]. Of the two articles that missed one or more estimand attributes, the first article did not state their approach to handle intercurrent events, and the second article did not specify how they would handle intercurrent events and failed to mention the summary measure to be reported (Table 4). Out of the five articles that contained an estimand, three (60%) had a composite outcome. Three (60%) articles stated the estimand in the main article, and 2 (40%) stated it in the appendices. One additional article did not specify a primary estimand but referenced the estimand framework in relation to a planned sensitivity analysis that would examine the impact of an intercurrent event [18].

**Table 3:** Specified attributes of the estimand in trials defining a primary estimand identified in review articles

Characteristics	No. of trials (*n* = 146)	%
‘Estimand’ term used		
No	140	96
Yes	6^*^	4
If stated where?		
Main article	4	67
Appendices	2	33
Is the primary estimand:		
Explicitly described (fully or partially)	5^*^	3
Not explicitly described	141	97
Population		
Stated	5	100
Not Stated	0	0
Treatment condition		
Stated	5	100
Not Stated	0	0
Outcome variable		
Stated	5	100
Not Stated	0	0
Outcome variable a composite variable incorporating or potentially incorporating an intercurrent event		
No	2	40
Yes	3	60
Handling of all relevant intercurrent events		
Stated	3	60
Not Stated	2	40
Strategy for handling intercurrent events		
Treatment policy	2	40
Hypothetical	1	20
Composite	2	40
While-on-treatment	0	0
Principal stratum	0	0
Other	0	0
Not applicable	0	0
Summary Measure		
Stated	4	80
Not Stated	1	20
Method of statistical analysis stated		
Stated	2	40
Not Stated	3	60

^*^One article did not specify their primary estimand but referenced the estimand framework in relation to a sensitivity analysis that would examine the impact of an intercurrent event

**Table 4:** Attributes of the estimand in trials defining a primary estimand by article

Primary author	Adaptive element	Intervention type	Article type	Attribute of estimand				
				Population	Treatment condition	Outcome variable	Handling of intercurrent events	Summary measure
Parker [14]	Multi-arm multi-stage platform	Drug	SAP	Stated	Stated	Stated	Stated	Stated
McLeod [15]	A multi-arm Bayesian adaptive randomised platform	Vaccine	Protocol	Stated	Stated	Stated	**Not stated**	**Not stated**
Stark [5]	Stopping rule	Transfusion	Protocol	Stated	Stated	Stated	Stated	Stated
Taselaar [16]	Sample size re-estimation	Nutritional	Protocol	Stated	Stated	Stated	**Not stated**	Stated
Francis [17]	Stopping rule	Drug	SAP	Stated	Stated	Stated	Stated	Stated

### Interim estimands

No SAPs or protocols stated the estimand for the interim analyses.

### Subgroup analyses

A larger percentage of SAPs (2/6, 33%) stated (fully or partially) their primary estimand compared to protocols (3/139, 2%) (Additional file 3: Appendix 4 Table 4). Of the 5 articles that stated a primary estimand (fully or partially), the two that did not state all five attributes were both protocols (Additional file 3: Appendix 4 Table 4).

Twenty percent (2/10) of articles with a pharmaceutical sponsor explicitly described their primary estimand (fully or partially) whilst only 3% (3/120) of articles with an academic sponsor stated an estimand (fully or partially) for the primary outcome (Additional file 3: Appendix 5 Table 4).

Both subgroup analyses contained very small sample sizes; of 146 articles included in this review, only six (4%) articles were SAPs, and only ten (7%) trials had a pharmaceutical sponsor. The full subgroup analyses are in Additional file 3: Appendices 4 and 5.

## Discussion

### Summary of findings

We found that only 3% of articles in this review specified their primary estimand. In addition, of the five articles we found that used the estimand framework, only three (2%) fully stated all five estimand attributes. Despite this being a marginal improvement since 2020 [7], the application of the estimand framework in RCT protocols and SAPs remains poor. We also found that no articles specified their estimand for the interim analyses or considered the implications of adaptations on their primary estimand.

Of the 3 articles stating their strategy for handling relevant intercurrent events, the majority used treatment policy or composite approaches. Due to the small number of articles, we are unable to draw wider conclusions on intercurrent strategies. These results should be interpreted with caution.

Two subgroup analyses were performed, one comparing SAPs and protocols and the other comparing articles with an academic sponsor to articles with a pharmaceutical sponsor. Overall, we found both SAPs and trials with a pharmaceutical sponsor were more likely to use the estimand framework to define their primary research question compared to protocols and trials with an academic sponsor. However, these subgroup results should be interpreted cautiously due to low numbers of SAPs and RCTs with pharmaceutical sponsors identified in this review.

### Implications

In order to more precisely describe the research questions being investigated, trialists need to implement the estimand framework and more transparently report both intercurrent events and strategies for handling them. The most appropriate strategy for handling intercurrent events will be context specific, depending on the study objectives and stakeholders. More consideration is needed to the question of interest and the impact of intercurrent events. Different strategies may be more appropriate or offer insights as an alternate strategy for supplementary analysis (which would require a secondary estimand).

Since completion of this review, the ICH E20 draft guidance on adaptive designs has been published [19]. With regards to estimands, this recognises the importance of pre-planning statistical methods for the primary analysis of adaptive trials aligned to each targeted estimand. It also acknowledges the use of adaptive trials with a planned selection of an estimand at an interim analysis, based on population or treatment selection, and notes that “all candidate estimands should be fully pre-specified and clinically relevant”. A commentary by Collignon et al. has previously discussed the importance of anticipating changes to the estimand that occur by design, e.g. adding new estimands in a platform trial as new interventions are introduced [20]. It also recognises how prespecified estimands can be selected following interim analysis, and that the trial continues with the remaining estimands. However, this nor the ICH E20 guidance explicitly makes any further recommendations on defining estimands for interim analysis. The most recent SPIRIT update (2025) now includes clear guidance on including trial objectives and acknowledges that estimands can aid clarity in this area in trial protocols, but again does not give guidance on estimands for interim analyses [21]. Researchers should describe estimands for both interim and final analysis, and where these differ the rationale and potential impact. This review emphasises the need for wider application of the estimand framework and clear guidance on implementation to trials with an adaptive design. Journal instructions to authors publishing protocols and SAPs with adaptive designs could also highlight the importance of specifying estimands for both interim and final analysis.

### Strengths and limitations

This review was conducted systematically, following a prespecified protocol. Screening and data extraction were piloted to ensure consistency across the reviewers.

Due to time and resource constraints, we only included two medical journals (BMJ Open and Trials). Including a wider range of medical journals would have given us a more representative sample; however, the chosen journals are known to publish a range of trial protocols and SAPs. A previous review examined the reporting and communication of sample size calculations in adaptive clinical trials identified trials via ClinicalTrials.gov and grant applications [22]. The authors found a greater number of industry-sponsored trials compared to public sector-sponsored trials (61.9% vs 38.1% respectively) in contrast to this review where trials were predominantly academic sponsored. This is likely to be a consequence of limiting our search to academic journal publications and the greater pressures within academic settings to publish. Therefore, the results and conclusions should be interpreted within the context of an academic setting.

Also due to the small number of reviewers, we individually screened and extracted data from the majority of articles. However, we piloted the data extraction process and double screened until consensus was reached for each stage of screening to ensure consistency across reviewers and double extracted information from the articles containing estimands.

This review was also limited to trials with an adaptive design; a broader review may have found greater improvements in the use of the estimand framework across trials more generally, but nonetheless gave valuable insights into the state of play of adaptive trials. Future work could also take a broader scope to better reflect both academic and industry-sponsored trials. Also, with time, it would be interesting to explore the impact of the recent publication of SPIRIT 2025 and the ICH E20 guidance [19, 21].

## Conclusions

Our review of RCT protocols and SAPs for adaptive trials highlights that trialists continue to not precisely define the research questions under investigation, making it difficult to understand the primary treatment effect of interest. We also found that the application of the estimand framework to interim analyses is non-existent and no consideration is being given to the impact of adaptations on their primary estimand; clear guidance is needed in this area.

## Supplementary Information

Additional file 1 (docx): Appendix 1. Review protocol. Appendix 2. Search strategy.

Additional file 2 (xlsm): Blank data extraction sheet.

Additional file 3 (docx): Appendix 3. Supplementary Results: Primary analysis. Appendix 4. Subgroup analysis: protocols vs SAPs. Appendix 5. Subgroup analysis: academic vs. pharmaceutical sponsor.

Additional file 4 (docx): PRISMA checklist. 

## Data Availability

The dataset used and analysed during the current study are available from the corresponding author on reasonable request.
