# Supplementary material for: Investigating estimand considerations in adaptive trials: a systematic review
Source: Trials. 2026 Feb 6;27:197. doi: 10.1186/s13063-026-09490-0 (PMC12973558; doi:10.1186/s13063-026-09490-0)
Supplement: Supplementary file 1 — Additional file 1 (docx): Appendix 1. Review protocol. Appendix 2. Search strategy. [file 13063_2026_9490_MOESM1_ESM.docx]

Additional file 1

Contents

[Appendix 1: Protocol 2](#_Toc196230379)

[Appendix 2: Search strategy 7](#_Toc196230380)

# Appendix 1: Protocol

**Review question**

Adaptive clinical trial designs allow researchers to make pre-planned changes to parts of the trial design once the study is underway, without undermining its validity and integrity (1). Such trials can allow for uncertainties at the point of design and are becoming more popular as they provide a more efficient means to establish if treatments are safe and effective (2).

Another area of focus in the clinical trials arena is the introduction of the estimand framework in the international trial regulatory guidelines (ICH E9(R1), 2019). This aims to ensure trialists precisely describe the research question a trial intends to investigate at the trial outset, by using estimands (3). The trial design, conduct and analysis can then be planned to align with the estimands of interest to ensure the trial addresses what is of most relevance.

A BMJ review of articles published in 2020 identified that the precise research question being investigated in most trials is unclear, mainly because of a lack of clarity on the approach to handling intercurrent (i.e. post-randomisation) events such as treatment withdrawal (4). Since then, the estimand framework has gained wider recognition.

This systematic review will build on the BMJ review to identify current application of the estimand framework specifically to adaptive clinical trials.

**Searches**

The search strategy will aim to identify published trial protocols and statistical analysis plans that describe adaptive randomised trials published in the year 2023. We will target two medical journals: the British Medical Journal (BMJ) Open and Trials that are known to publish articles describing trial protocols and statistical analysis plans.

The search will be performed using the MEDLINE and EMBASE databases (access via Ovid).

**Types of study to be included**

Adaptive randomised controlled trials

**Condition or domain being studied**

Treatment estimands in the statistical analysis of adaptive randomised controlled trials, with no restrictions on medical conditions.

Adaptive design randomised controlled trials are defined as outlining pre-planned modifications to the trial.

**Participants/population**

Inclusion criteria:

Full text protocols, or statistical analysis plans, published in either BMJ Open or Trials describing adaptive design randomised controlled trial in humans in the English language

Exclusion criteria:

- Pilot or feasibility trial protocol or statistical analysis plans
- Phase I protocol or statistical analysis plans
- Protocol or statistical analysis plans for non-randomised designs
- Protocol or statistical analysis plans for meta-analyses and/or systematic reviews
- Articles describing the analysis of a randomised controlled trial
- Cost-effectiveness as primary outcome
- Letters/commentaries

**Intervention(s), exposure(s)**

Include all eligible adaptive designed randomised trials, regardless of the intervention.

**Comparator(s)/control**

Include all eligible adaptive designed randomised trials, regardless of the comparator.

**Main outcome(s)**

1. How often the primary estimand is:
2. explicitly described, or
3. not explicitly described?
4. If interim analyses are planned how often the estimand for the interim analyses is:

All 5 elements completely stated or not stated

1. How often intercurrent events (one or more) are described?

Explicitly, not explicitly, no

**Data extraction (selection and coding)**

All screening for eligibility and data extraction process will be conducted using Covidence web-based tool. Search results from across individual databases will be imported into Covidence and duplicates removed. Two reviewers will screen the titles and abstracts identified from the search based on inclusion/exclusion criteria.

In the first instance, the screening of the titles and abstracts, against the pre-defined inclusion/exclusion criteria, will be carried out by two reviewers. Dependent on the number of titles and abstracts, a random sample of 5% or 20 titles (whichever is smaller) will be screened and compared across reviewers to ensure consensus. If there are disagreements a larger random sample will be compared until there is confidence in accurate screening. After screening the remaining titles and abstracts will be shared between both reviewers for screening to form a full extract list.

The extract list will form the articles that will be fully screened against the pre-defined inclusion/exclusion criteria. Again, dependent on the number of full articles, a random sample of 5% or 10 titles (whichever is smaller) will be screened and compared across reviewers to ensure consensus. If there are disagreements a larger random sample will be compared until there is confidence in accurate screening. After screening the remaining full articles will be shared between both reviewers for screening to form a final list.

The final list will form the articles that will undergo data extraction. Data from the full-text articles will be extracted by a sole reviewer onto an initial pre-defined data extraction template. Full-text articles that do not fulfil the inclusion/exclusion criteria will not undergo data extraction. The pre-defined data extraction template will be used to extract data from 5% or 10 articles, whichever is smaller. This will then be reviewed by all collaborators to determine if the data extract form is sufficient. Once agreement on the final data extract form is reached, the remaining full articles will undergo data extraction.

Any uncertainties will be resolved by consensus between the two reviewers in the first instance, and where consensus is not reached, the decision will be done with a third reviewer. If necessary, the inclusion criteria will be modified or expanded to remove any ambiguity.

Extracted data fields will include:

**Trial characteristics**:

- Author
- Journal
- Sponsor [pharmaceutical or for-profit/academic or not for profit/unclear
- Type of intervention [Drug/Vaccine/Surgery/Nutritional/Behavioural/Digital/Other]
- Intervention delivery [one-off/multiple short-term (≤1 week)/multiple mid-term (≤ 1 month)/multiple long-term (>1 month)]

**Design characteristics**:

- Planned sample size
- Planned number of trial arms
- Adaptive elements e.g. GSD, sample size re-estimation etc
  - For each adaptive element describe e.g. For GSD type of stopping rule used, number of interim analyses etc

**Relevant intercurrent events**:

- Whether any intercurrent events are described, even if not explicitly identified as an intercurrent event
- The type(s) of intercurrent events described or reported, classified as:
  - Treatment non-adherence with no reason
  - Treatment non-adherence due to AE
  - Treatment non-adherence with reason (not AE)
  - Treatment discontinuation with no reason
  - Treatment discontinuation due to AE
  - Treatment discontinuation with reason (not AE)
  - Use of additional treatment not part of usual care (e.g. rescue therapy)
  - Treatment switching
  - Death
  - Other

**Primary estimand**:

- ICH-E9 addendum referenced
- ‘Estimand’ term used
- Population [stated / not stated]
- Population details
- Treatment condition(s) [stated / not stated]
- Treatment condition(s) details
- Outcome variable (or endpoint) [stated/ not stated]
- Outcome variable type
- Outcome variable a composite variable incorporating or potentially incorporating an intercurrent event
- Handling of all relevant intercurrent events [stated/not stated]
- Strategy for handling intercurrent events [treatment policy/hypothetical/composite/while-on-treatment/principal stratum/other]
- Population level summary [stated/not stated]
- Population level summary details
- Method of statistical analysis and stated assumptions if non-treatment policy strategy used

**Estimand for interim analysis**:

- Population [stated/not stated]
- Population details
- Treatment condition(s) [stated/not stated]
- Treatment condition(s) details
- Outcome variable (or endpoint) [stated/not stated]
- Outcome variable type
- Handling of all relevant intercurrent events [stated/inferable/unclear/no event occurrences reported]
- Strategy for handling intercurrent events [treatment policy/hypothetical/composite/while-on-treatment/principal stratum/other]
- Population level summary [stated/not stated]
- Population level summary details
- Method of statistical analysis and stated assumptions if non-treatment policy strategy used

**Where details are published:** Main article or Appendices

**Risk of bias assessment**

This is a review of estimands, intercurrent events and the strategies and statistical methods used to handle intercurrent events in adaptive randomised trials. As a result we will not be undertaking a risk of bias evaluation.

**Strategy for data synthesis**

Data will be imported into a statistical software package such as Stata or R for analysis. All outcomes described above will be summarised descriptively in tabular form.

We will establish how well estimands (primary, supplementary and interim) are described overall, using the extracted data on the 5 estimand attributes (population, treatment condition(s), outcome variable, handling of intercurrent events and population level summary). If all five attributes are recorded as ‘stated’ then the overall estimand will be stated.

Separate summary tables will describe (i) trial characteristics, (ii) intercurrent events, (iii) the description and detail of primary trial estimands (iv), the description and detail of trial estimands for the interim analysis. Frequencies and percentages will be used to summarise categorical outcomes. Continuous variables will be summarised using mean (standard deviation) if approximately normally distributed or median (Interquartile range) if skewed.

This review will extract estimand definitions and details on the statistical methods proposed for estimation in trial protocols and statistical analysis plans. This will result in a directory of protocols and statistical analysis plans that use the estimand framework in adaptive trials which will be made publicly available.

**Analysis of subgroups or subsets**

Planned subgroup analysis involved comparing

- Sponsorship: academic/not-for-profit or pharmaceutical/for-profit sponsors.
- Article type: SAP or protocol.

**References**

1. Mahajan R, Gupta K. Adaptive design clinical trials: Methodology, challenges and prospect. Indian J Pharmacol. 2010;42(4):201-7.

2. Collignon O, Koenig F, Koch A, Hemmings RJ, Pétavy F, Saint-Raymond A, et al. Adaptive designs in clinical trials: from scientific advice to marketing authorisation to the European Medicine Agency. Trials. 2018;19(1):642.

3. ICH E9 (R1) Addendum on Estimands and Sensitivity Analysis in Clinical Trials to the Guideline on Statistical Principles for Clinical Trials, (2019).

4. Cro S, Kahan BC, Rehal S, Chis Ster A, Carpenter JR, White IR, et al. Evaluating how clear the questions being investigated in randomised trials are: systematic review of estimands. BMJ. 2022;378:e070146.

# Appendix 2: Search strategy

*Ovid MEDLINE(R)*

| 1 | exp Randomized Controlled Trial/ |
| --- | --- |
| 2 | controlled clinical trial.pt. |
| 3 | randomized.ab. |
| 4 | randomised.ab. |
| 5 | placebo.ab. |
| 6 | Clinical trials as topic/ |
| 7 | randomly.ab. |
| 8 | trial.ti |
| 9 | or/1-8 |
| 10 | exp animals/ not humans/ |
| 11 | 9 not 10 |
| 12 | trials electronic resource.jn. |
| 13 | bmj open.jn. |
| 14 | 12 or 13 |
| 15 | (protocol or statistical analysis plan or SAP).mp. |
| 16 | (systematic review or meta-analysis).m_titl. |
| 17 | 11 and 14 and 15 |
| 18 | 17 not 16 |
| 19 | limit 18 to yr=”2023” |

Titles: 1293

*Ovid EMBASE*

| 1 | exp randomized controlled trial/ |
| --- | --- |
| 2 | controlled clinical trial.de. |
| 3 | randomized.ab. |
| 4 | randomised.ab. |
| 5 | placebo.ab. |
| 6 | “clinical trial (topic)”/ |
| 7 | randomly.ab. |
| 8 | trial.ti |
| 9 | or/1-8 |
| 10 | exp animal/ not exp human/ |
| 11 | 9 not 10 |
| 12 | trials.jn. |
| 13 | bmj open.jn. |
| 14 | 12 or 13 |
| 15 | (protocol or statistical analysis plan or SAP).mp. |
| 16 | (systematic review or meta-analysis).m_titl. |
| 17 | 11 and 14 and 15 |
| 18 | 17 not 16 |
| 19 | limit 18 to yr=”2023” |

Titles: 1354
