# Supplementary material for: Investigating estimand considerations in adaptive trials: a systematic review
Source: Trials. 2026 Feb 6;27:197. doi: 10.1186/s13063-026-09490-0 (PMC12973558; doi:10.1186/s13063-026-09490-0)
Supplement: Supplementary file 3 — Additional file 3 (docx): Appendix 3. Supplementary Results: Primary analysis. Appendix 4. Subgroup analysis: protocols vs SAPs. Appendix 5. Subgroup analysis: academic vs. pharmaceutical sponsor. [file 13063_2026_9490_MOESM3_ESM.docx]

Additional file 3

Contents

[Appendix 3: Supplementary Results: Primary analysis 2](#_Toc197531334)

[Appendix 4: Subgroup analysis: protocols vs SAPs* 8](#_Toc197531335)

[Appendix 5: Subgroup analysis: academic vs pharmaceutical sponsor* 14](#_Toc197531336)

# Appendix 3: Supplementary Results: Primary analysis

| Table 1: Article Details | | |
| --- | --- | --- |
| **Characteristics** | **No. of Trials**  **(n=146)** | **%** |
| Journal | | |
| Trials | 86 | 59 |
| BMJ Open | 60 | 41 |
| Sponsor | | |
| Pharmaceutical or for-profit | 10 | 7 |
| Academic or not for profit | 120 | 82 |
| Unclear | 16 | 11 |
| Type | | |
| SAP | 6 | 4 |
| Protocol | 139 | 95 |
| SAP & Protocol | 1 | 1 |

Acronym: SAP – statistical analysis plan

| Table 2: Trial Details | | |
| --- | --- | --- |
| **Characteristics** | **No. of Trials**  **(n=146)** | **%** |
| Primary country | | |
| Australia | 10 | 7 |
| Canada | 10 | 7 |
| China | 28 | 19 |
| Germany | 4 | 3 |
| Italy | 4 | 3 |
| Mali | 3 | 2 |
| Netherlands | 9 | 6 |
| Spain | 4 | 3 |
| UK | 11 | 8 |
| USA | 22 | 15 |
| Other | 41 | 28 |
| Site | | |
| Multi-centre | 99 | 68 |
| Single-centre | 42 | 29 |
| Unclear | 5 | 3 |
| Type of Intervention: | | |
| Behavioural | 11 | 8 |
| Digital | 9 | 6 |
| Drug | 48 | 33 |
| Medical device | 10 | 7 |
| Nutritional | 7 | 5 |
| Other | 39 | 27 |
| Surgery | 21 | 14 |
| Vaccine | 1 | 1 |
| Intervention delivery | | |
| Multiple long-term (>1 month) | 55 | 38 |
| Multiple mid-term (≤ 1 month) | 26 | 18 |
| Multiple short-term (≤1 week) | 18 | 12 |
| One-off | 45 | 31 |
| Unclear | 2 | 1 |
| Planned sample size | | |
| Median (IQR) | 365 | (168, 1000) |
| Planned number of arms (including control) | | |
| 2 | 121 | 83 |
| 3 | 12 | 8 |
| 4 | 9 | 6 |
| 13 | 1 | 1 |
| Unclear | 3 | 2 |
| Planned number of interims | | |
| 1 | 91 | 62 |
| 2 | 18 | 12 |
| 3 | 10 | 7 |
| 4 | 1 | 1 |
| 6 | 1 | 1 |
| Unclear | 25 | 17 |
| Planned addition or dropping of arms | | |
| No | 138 | 95 |
| Yes | 8 | 5 |
| Planned stopping rule |  |  |
| No | 32 | 22 |
| Yes | 116 | 78 |
| Planned sample size re-estimation | | |
| No | 113 | 77 |
| Yes | 33 | 23 |
| Planned adaptive randomisation | | |
| No | 134 | 92 |
| Yes | 12 | 8 |

Appendix 4: Subgroup analysis: protocols vs SAPs*

| Table 1: Article Details: Protocols vs SAPs | | | | |
| --- | --- | --- | --- | --- |
| **Characteristics** | **Protocols**  **(n=139)** | | **SAPs**  **(n=6)** | |
|  | **No. of Trials** | **%** | **No. of Trials** | **%** |
| Journal | | | | |
| Trials | 79 | 57 | 6 | 100 |
| BMJ Open | 60 | 43 | 0 | 0 |
| Sponsor | | | | |
| Pharmaceutical or for-profit | 9 | 6 | 1 | 17 |
| Academic or not for profit | 114 | 82 | 5 | 83 |
| Unclear | 16 | 12 | 0 | 0 |

*One article was both a protocol and SAP combined and has been omitted from this table (n=145)

| Table 2: Trial Details: Protocols vs SAPs* | | | | |
| --- | --- | --- | --- | --- |
|  | **Protocols**  **(n=139)** | | **SAPs**  **(n=6)** | |
| **Characteristics** | **No. of Trials** | **%** | **No. of Trials** | **%** |
| Primary country |  |  |  |  |
| Australia | 8 | 6 | 2 | 33 |
| Canada | 9 | 6 | 1 | 17 |
| China | 28 | 20 | 0 | 0 |
| Germany | 4 | 3 | 0 | 0 |
| Italy | 4 | 3 | 0 | 0 |
| Mali | 1 | 1 | 2 | 33 |
| Netherlands | 9 | 6 | 0 | 0 |
| Spain | 4 | 3 |  |  |
| UK | 10 | 7 | 1 | 17 |
| USA | 21 | 15 | 0 | 0 |
| Other | 41 | 29 | 0 | 0 |
| Site | | |  |  |
| Multi-centre | 93 | 67 | 6 | 100 |
| Single-centre | 41 | 29 | 0 | 0 |
| Unclear | 5 | 4 | 0 | 0 |
| Type of Intervention: | | |  |  |
| Behavioural | 11 | 8 | 0 | 0 |
| Digital | 9 | 6 | 0 | 0 |
| Drug | 43 | 31 | 4 | 67 |
| Medical device | 10 | 7 | 0 | 0 |
| Nutritional | 7 | 5 | 0 | 0 |
| Other | 38 | 27 | 1 | 17 |
| Surgery | 20 | 14 | 1 | 17 |
| Vaccine | 1 | 1 | 0 | 0 |
| Intervention delivery | | |  |  |
| Multiple long-term (>1 month) | 52 | 37 | 3 | 50 |
| Multiple mid-term (≤ 1 month) | 25 | 18 | 0 | 0 |
| Multiple short-term (≤1 week) | 17 | 12 | 1 | 17 |
| One-off | 44 | 32 | 1 | 17 |
| Unclear | 1 | 1 | 1 | 17 |
| Planned sample size | | |  |  |
| Median (IQR) | 360 | (166, 875) | 1010 | (531, 1151) |
| Planned number of arms (including control) | | |  |  |
| 2 | 116 | 83 | 4 | 67 |
| 3 | 10 | 7 | 2 | 33 |
| 4 | 9 | 6 | 0 | 0 |
| 13 | 1 | 1 | 0 | 0 |
| Unclear | 3 | 1 | 0 | 0 |
| Planned number of interims | | |  |  |
| 1 | 88 | 63 | 2 | 33 |
| 2 | 15 | 11 | 3 | 50 |
| 3 | 9 | 6 | 1 | 17 |
| 4 | 1 | 1 | 0 | 0 |
| 6 | 1 | 1 | 0 | 0 |
| Unclear | 25 | 18 | 0 | 0 |
| Planned addition or planned dropping of arms | | |  | |
| No | 133 | 96 | 4 | 67 |
| Yes | 6 | 4 | 2 | 33 |
| Planned stopping rule | | |  | |
| No | 32 | 23 | 0 | 0 |
| Yes | 107 | 77 | 6 | 100 |
| Planned sample size re-estimation | | |  | |
| No | 106 | 77 | 6 | 0 |
| Yes | 33 | 24 | 0 | 100 |
| Planned adaptive randomisation | | |  | |
| No | 127 | 92 | 6 | 0 |
| Yes | 12 | 9 | 0 | 100 |

*One article was both a protocol and SAP combined and has been omitted from this table (n=145)

| Table 3: Intercurrent Events: Protocols vs SAPs* | | | | |
| --- | --- | --- | --- | --- |
| **Characteristics** | **Protocols**  **(n=139)** | | **SAPs**  **(n=6)** | |
|  | **No. of Trials** | **%** | **No. of Trials** | **%** |
| Intercurrent event described | | |  |  |
| No | 49 | 35 | 3 | 50 |
| Yes explicitly | 3 | 2 | 2 | 33 |
| Yes, not explicitly | 87 | 63 | 1 | 17 |
| Type of intercurrent event described | | | | |
| Treatment non-adherence with no reason | 21 | 10 | 1 | 5 |
| Treatment non-adherence due to AE | 22 | 11 | 3 | 17 |
| Treatment non-adherence with reason (not AE) | 25 | 12 | 3 | 17 |
| Treatment discontinuation with no reason | 37 | 18 | 3 | 17 |
| Treatment discontinuation due to AE | 44 | 21 | 2 | 11 |
| Treatment discontinuation with reason (not AE) | 36 | 17 | 2 | 11 |
| Use of additional treatment not part of usual care (e.g. rescue therapy) | 7 | 3 | 1 | 5 |
| Treatment switching | 2 | 1 | 1 | 5 |
| Death | 13 | 6 | 2 | 11 |
| Other | 2 | 11 | 0 | 0 |

*One article was both a protocol and SAP combined and has been omitted from this table (n=145)

| Table 4: Primary Estimand: Protocols vs SAPs* | | | | |
| --- | --- | --- | --- | --- |
| **Characteristics** | **Protocols**  **(n=139)** | | **SAPs**  **(n=6)** | |
|  | **No. of Trials** | **%** | **No. of Trials** | **%** |
| ‘Estimand’ term used | | | | |
| No | 135 | 97 | 4 | 67 |
| Yes** | 4 | 3 | 2 | 33 |
| If stated where? | | | | |
| Main article | 2 | 50 | 2 | 100 |
| Appendices | 2 | 50 | 0 | 0 |
| Is the primary estimand: | | | | |
| Explicitly described (fully or partially)** | 3 | 2 | 2 | 33 |
| Not explicitly described | 136 | 98 | 4 | 67 |
| Population | | | | |
| Stated | 3 | 100 | 2 | 100 |
| Not Stated | 0 | 0 | 0 | 0 |
| Treatment condition | | | | |
| Stated | 3 | 100 | 2 | 100 |
| Not Stated | 0 | 0 | 0 | 0 |
| Outcome variable | | | | |
| Stated | 3 | 100 | 2 | 100 |
| Not Stated | 0 | 0 | 0 | 0 |
| Outcome variable a composite variable incorporating or potentially incorporating an intercurrent event | | | | |
| No | 2 | 67 | 0 | 0 |
| Yes | 1 | 33 | 2 | 100 |
| Handling of all relevant intercurrent events | | | | |
| Stated | 1 | 33 | 2 | 100 |
| Not Stated | 2 | 67 | 0 | 0 |
| Strategy for handling intercurrent events | | | | |
| Treatment policy | 1 | 50 | 1 | 33 |
| Hypothetical | 0 | 0 | 1 | 33 |
| Composite | 1 | 50 | 1 | 33 |
| While-on-treatment | 0 | 0 | 0 | 0 |
| Principal stratum | 0 | 0 | 0 | 0 |
| Other | 0 | 0 | 0 | 0 |
| NA | 0 | 0 | 0 | 0 |
| Summary Measure | | | | |
| Stated | 2 | 67 | 2 | 100 |
| Not Stated | 1 | 33 | 0 | 0 |
| Method of statistical analysis and stated | | | | |
| Stated | 1 | 33 | 1 | 50 |
| Not Stated | 2 | 67 | 1 | 50 |

*One article was both a protocol and SAP combined and has been omitted from this table (n=145)

**One article did not specify their primary estimand but referenced the estimand framework in relation to a sensitivity analysis that would examine the impact of an intercurrent event

Appendix 5: Subgroup analysis: academic vs pharmaceutical sponsor*

| Table 1: Article Details: Academic vs Pharmaceutical Sponsor | | | | |
| --- | --- | --- | --- | --- |
| **Characteristics** | **Academic**  **(n=120)** | | **Pharmaceutical**  **(n=10)** | |
|  | **No. of Trials** | **%** | **No. of Trials** | **%** |
| Journal | | | | |
| Trials | 77 | 64 | 4 | 40 |
| BMJ Open | 43 | 36 | 6 | 60 |
| Type | | | | |
| SAP | 5 | 4 | 1 | 10 |
| Protocol | 114 | 95 | 9 | 90 |
| SAP & Protocol | 1 | 1 | 0 | 0 |

*Sixteen articles sponsor was unclear (n=130)

| Table 2: Trial Details: Academic vs Pharmaceutical Sponsor* | | | | |
| --- | --- | --- | --- | --- |
|  | **Academic**  **(n=120)** | | **Pharmaceutical**  **(n=10)** | |
| **Characteristics** | **No. of Trials** | **%** | **No. of Trials** | **%** |
| Primary country | | |  | |
| Australia | 9 | 8 | 1 | 10 |
| Canada | 9 | 8 | 0 | 0 |
| China | 21 | 18 | 1 | 10 |
| Germany | 3 | 3 | 1 | 10 |
| Italy | 3 | 3 | 1 | 10 |
| Mali | 3 | 3 | 0 | 0 |
| Netherlands | 5 | 4 | 3 | 30 |
| Spain | 3 | 3 | 1 | 10 |
| UK | 11 | 9 | 0 | 0 |
| USA | 18 | 15 | 2 | 20 |
| Other | 35 | 29 | 0 | 0 |
| Site | | |  |  |
| Multi-centre | 84 | 70 | 7 | 70 |
| Single-centre | 33 | 28 | 3 | 30 |
| Unclear | 3 | 3 | 0 | 0 |
| Type of Intervention: | | |  |  |
| Behavioural | 9 | 8 | 0 | 0 |
| Digital | 8 | 7 | 0 | 0 |
| Drug | 40 | 33 | 3 | 30 |
| Medical device | 8 | 7 | 1 | 10 |
| Nutritional | 4 | 3 | 2 | 20 |
| Other | 32 | 27 | 2 | 20 |
| Surgery | 18 | 15 | 2 | 20 |
| Vaccine | 1 | 1 | 0 | 0 |
| Intervention delivery | | |  | |
| Multiple long-term (>1 month) | 47 | 39 | 3 | 30 |
| Multiple mid-term (≤ 1 month) | 21 | 18 | 1 | 10 |
| Multiple short-term (≤1 week) | 11 | 9 | 3 | 30 |
| One-off | 39 | 33 | 3 | 30 |
| Unclear | 2 | 2 | 0 | 0 |
| Planned sample size | | |  | |
| Median (IQR) | 362 | (168, 1000) | 809 | (292, 1315) |
| Planned number of arms (including control) | | |  | |
| 2 | 98 | 82 | 10 | 100 |
| 3 | 9 | 8 | 0 | 0 |
| 4 | 9 | 8 | 0 | 0 |
| 13 | 1 | 1 | 0 | 0 |
| Unclear | 3 | 3 | 0 | 0 |
| Planned number of interims | | |  | |
| 1 | 70 | 58 | 6 | 60 |
| 2 | 17 | 14 | 1 | 10 |
| 3 | 7 | 6 | 2 | 20 |
| 4 | 0 | 0 | 1 | 10 |
| 6 | 1 | 1 | 0 | 0 |
| Unclear | 25 | 21 | 0 | 0 |
| Planned addition or planned dropping of arms | | |  | |
| No | 112 | 93 | 10 | 100 |
| Yes | 8 | 7 | 0 | 0 |
| Planned stopping rule | | |  | |
| No | 25 | 21 | 2 | 20 |
| Yes | 95 | 79 | 8 | 80 |
| Planned sample size re-estimation | | |  | |
| No | 96 | 80 | 6 | 60 |
| Yes | 24 | 20 | 4 | 40 |
| Planned adaptive randomisation | | |  | |
| No | 110 | 92 | 10 | 100 |
| Yes | 10 | 8 | 0 | 100 |

*Sixteen articles sponsor was unclear (n=130)

| Table 3: Intercurrent Events: Academic vs Pharmaceutical Sponsor* | | | | |
| --- | --- | --- | --- | --- |
| **Characteristics** | **Academic**  **(n=120)** | | **Pharmaceutical**  **(n=10)** | |
|  | **No. of Trials** | **%** | **No. of Trials** | **%** |
| Intercurrent event described | | |  |  |
| No | 41 | 34 | 3 | 30 |
| Yes explicitly | 3 | 3 | 1 | 10 |
| Yes, not explicitly | 76 | 63 | 6 | 60 |
| Type of Intercurrent Event Described | | | | |
| Treatment non-adherence with no reason | 18 | 9 | 2 | 13 |
| Treatment non-adherence due to AE | 24 | 12 | 2 | 13 |
| Treatment non-adherence with reason (not AE) | 25 | 12 | 2 | 13 |
| Treatment discontinuation with no reason | 35 | 17 | 4 | 25 |
| Treatment discontinuation due to AE | 43 | 21 | 1 | 6 |
| Treatment discontinuation with reason (not AE) | 34 | 17 | 3 | 19 |
| Use of additional treatment not part of usual care (e.g. rescue therapy) | 7 | 3 | 1 | 6 |
| Treatment switching | 3 | 1 | 0 | 0 |
| Death | 11 | 5 | 1 | 6 |
| Other | 2 | 1 | 0 | 0 |

| Table 4: Primary Estimand: Academic vs Pharmaceutical Sponsor* | | | | |
| --- | --- | --- | --- | --- |
| **Characteristics** | **Academic**  **(n=120)** | | **Pharmaceutical**  **(n=10)** | |
|  | **No. of Trials** | **%** | **No. of Trials** | **%** |
| ‘Estimand’ term used | | | | |
| No | 116 | 97 | 8 | 80 |
| Yes | 4 | 3 | 2 | 20 |
| If stated where? | | | | |
| Main article | 2 | 67 | 1 | 50 |
| Appendices | 1 | 33 | 1 | 50 |
| Is the primary estimand: | | | | |
| Explicitly described | 3 | 3 | 2 | 20 |
| Not explicitly described | 117 | 98 | 8 | 80 |
| Population | | | | |
| Stated | 3 | 100 | 2 | 100 |
| Not Stated | 0 | 0 | 0 | 0 |
| Treatment condition | | | | |
| Stated | 3 | 100 | 2 | 100 |
| Not Stated | 0 | 0 | 0 | 0 |
| Outcome variable | | | | |
| Stated | 3 | 100 | 2 | 100 |
| Not Stated | 0 | 0 | 0 | 0 |
| Outcome variable a composite variable incorporating or potentially incorporating an intercurrent event | | | | |
| No | 1 | 33 | 1 | 50 |
| Yes | 2 | 67 | 1 | 50 |
| Handling of all relevant intercurrent events | | | | |
| Stated | 2 | 67 | 1 | 50 |
| Not Stated | 1 | 33 | 1 | 50 |
| Strategy for handling intercurrent events | | | | |
| Treatment policy | 2 | 50 | 0 | 0 |
| Hypothetical | 1 | 25 | 0 | 0 |
| Composite | 1 | 25 | 1 | 100 |
| While-on-treatment | 0 | 0 | 0 | 0 |
| Principal stratum | 0 | 0 | 0 | 0 |
| Other | 0 | 0 | 0 | 0 |
| NA | 0 | 0 | 0 | 0 |
| Summary Measure | | | | |
| Stated | 2 | 67 | 0 | 0 |
| Not Stated | 1 | 33 | 2 | 100 |
| Method of statistical analysis and stated | | | | |
| Stated | 2 | 67 | 0 | 0 |
| Not Stated | 1 | 33 | 2 | 100 |

*Sixteen articles sponsor was unclear (n=130)
